# Pawlak Algebra and Approximate Structure on Fuzzy Lattice

**DOI:** 10.1155/2014/697107

**Published:** 2014-07-23

**Authors:** Ying Zhuang, Wenqi Liu, Chin-Chia Wu, Jinhai Li

**Affiliations:** ^1^Faculty of Science, Kunming University of Science and Technology, Kunming 650500, China; ^2^Department of Statistics, Feng Chia University, Taichung 40724, Taiwan

## Abstract

The aim of this paper is to investigate the general approximation structure, weak approximation operators, and Pawlak algebra in the framework of fuzzy lattice, lattice topology, and auxiliary ordering. First, we prove that the weak approximation operator space forms a complete distributive lattice. Then we study the properties of transitive closure of approximation operators and apply them to rough set theory. We also investigate molecule Pawlak algebra and obtain some related properties.

## 1. Introduction

The theory of rough sets was originally proposed by Pawlak [1] in 1982 as a mathematical approach to handle imprecision, vagueness, and uncertainty in data analysis, which has been applied successfully to many areas such as knowledge representation, data mining, pattern recognition, and decision making (Sun et al. [2], Wang et al. [3]). This theory takes into consideration the indiscernibility between objects. The indiscernibility is typically characterized by an equivalence relation. Rough sets are the results of approximating crisp sets using equivalence classes. However, the requirement of an equivalence relation seems to be a very restrictive condition which may limit the applications of rough set theory since this requirement can deal only with complete information systems. Therefore, some interesting and meaningful extensions of Pawlak's rough set models have been proposed in the literature. For example, some interesting extensions to equivalence relations have been proposed, such as tolerance relations or similarity relations, general binary relations on the discourse, partitions and general binary relations on the neighborhood system from topological space, and general approximation spaces (examples of this approach can be found in Chen et al. [4], Wang and Hu [5], and Yin et al. [6–8]); some general notions of rough sets such as rough fuzzy sets, fuzzy rough sets, and soft rough sets have been proposed and discussed (examples of this approach can be found in Ali et al. [9], Feng et al. [10], Li and Yin [11], Yao et al. [12], and Zhang et al. [13]); and rough set models on two universes of discourse which can be interpreted by the notions of interval structure and generalized approximation space have been extensively studied by Li and Zhang [14], Ma and Sun [15], and Yao and Lin [16].

The relationships between lattice theory and rough sets are another topic receiving much attention in recent years. Cattaneo and Ciucci [17] focus on the study on lattices with interior and closure operators and abstract approximation spaces, in which the nonequational notion of abstract approximation space for roughness theory is introduced, and its relationship with the equational definition of lattice with Tarski interior and closure operations is studied. Their categorical isomorphism is also proved, and the role of the Tarski interior and closure with an algebraic semantic of a S4-like model of modal logic is widely investigated. Järvinen [18] investigates lattice-theoretical foundations of rough set theory, in which closure operators in a more general setting of ordered sets, fixpoints of Galois connections, rough set approximations and definable sets, and the lattice structures of the ordered set of all rough sets determined by different kinds of indiscernibility relations are studied in detail. The purpose of this paper is to investigate the general approximation structure, weak approximation operators, and Pawlak algebra in the framework of fuzzy lattice, lattice topology, and auxiliary ordering. The relationships between the Pawlak approximation structures and these mathematic structures are established.

The remaining part of the paper is organized as follows.  Section 2 introduces the relevant definitions which will be used throughout the paper.  Section 3 investigates the Pawlak algebra and weak Pawlak algebra on fuzzy lattice.  Section 4 discusses the relationships between Pawlak algebra and auxiliary ordering.  Section 5 focuses on the study of molecular Pawlak algebra.  Section 6 investigates the properties of Pawlak algebra based on binary relation. Finally, Section 7 concludes the paper and suggests some future research topics.

## 2. Preliminaries

In this section, we introduce some basic definitions (see [3, 19, 20]) which will be used throughout the paper.

**Definition 1 .**

A partially ordered set (*L*, ≤) is said to be a lattice if inf⁡⁡{*x*, *y*} and sup⁡⁡{*x*, *y*}, denoted by ∧ and ∨, respectively, exist, for all *x*, *y* ∈ *L*. A lattice *L* is said to be* complete* if, for every *A*⊆*L*, ⋀_*α*∈*A*_ *α* and ⋁_*α*∈*A*_ *α* exist.

**Definition 2 .**

Let (*L*, ≤) be a complete lattice with the maximum element 1 and minimum element 0 and “≺” a binary relation on *L*. If the following conditions hold: for all *α*, *β*, *μ*, *η*, *γ* ∈ *L*,

- *α*≺*β*⇒*α* ≤ *β*,
- *μ* ≤ *α*≺*β* ≤ *η*⇒*μ*≺*η*,
- *α*≺*γ*, *β*≺*γ*⇒*α*∨*β*≺*γ*,
- ∀*α* ∈ *L*, 0≺*α*≺1,

then the relation “≺” is called an* auxiliary ordering* on *L*. If the relation “≺” satisfies conditions (i), (ii), and (iv), it is called a* weak auxiliary ordering* on *L*. The weak auxiliary ordering “≺” is called* completely approximate* if

(1) $$\begin{array}{c} \forall \alpha \in L,\;\alpha =\vee \left\{\gamma \;|\;\gamma ≺\alpha ,\;\;\gamma \in L\right\}. \end{array}$$

Furthermore, if the relation “≺” satisfies conditions (i), (ii), (iv), and

(iii)′ *α*_*t*_≺*β*(*t* ∈ *T*)⇒⋁_*t*∈*T*_ *α*_*t*_≺*β*,

then “≺” is called a strong auxiliary ordering on *L*.

**Definition 3 .**

Let (*L*, ∨, ∧, 0,1) be a completely distributive lattice. If the mapping ^*c*^ : *L* → *L* satisfies the following conditions:

- reverse law: ∀*α*, *β* ∈ *L*, if *α* ≤ *β*, then *β* ^*c*^ ≤ *α* ^*c*^,
- recovery law: ∀*α* ∈ *L*, (*α* ^*c*^)^*c*^ = *α*,

then (*L*, ∨, ∧,^*c*^, 0,1) is called a* fuzzy lattice*.

**Remark 4 .**

The notion of fuzzy lattice just given is first introduced in Wang [20] and is also called de Morgan completely distributive lattice in the literature. To keep consistency, we adopt the term “fuzzy lattice” in this paper.

**Definition 5 .**

Let (*L*, ∨, ∧,^*c*^, 0,1) be a fuzzy lattice and *π*⊆*L*. If the subset *π* satisfies the following conditions:

- 0 ∉ *π*,
- *α*, *β* ∈ *π*, *α*∧*β* ≠ 0⇒*α* ≤ *β*  or  *β* ≤ *α*,
- *α*, *β*, *γ* ∈ *π*, *α* ≤ *γ*, *β* ≤ *γ*⇒*α*∧*β* ≠ 0,
- *η* = ∨{*α*∣*α* ∈ *π*, *α* ≤ *η*} for all *η* ∈ *L*,
- if *π*_0_⊆*π* and *π*_0_ is linear order subset, then ∨*π*_0_ ∈ *π*,

then *π* is called a* molecular set*, and the element of *π* is called a* molecule*. The fuzzy lattice with molecule (*L*(*π*), ∨, ∧,^*c*^, 0,1) is called a* molecular lattice*.

**Definition 6 .**

Let (*L*, ∨, ∧,^*c*^, 0,1) be a fuzzy lattice and *δ*⊆*L*. If the subset *δ* satisfies the following conditions:

1. 0, 1 ∈ *δ*,
2. *α*_*t*_ ∈ *δ*(*t* ∈ *T*)⇒⋁_*t*∈*T*_ *α*_*t*_ ∈ *δ*,
3. *α*, *β* ∈ *δ*⇒*α*∧*β* ∈ *δ*,

then (*L*, *δ*) is called a lattice topology space.

## 3. The Pawlak Algebra and Weak Pawlak Algebra on Fuzzy Lattices

In this section, we investigate the structural properties of Pawlak rough approximations on fuzzy lattices. Let us begin with introducing the following concepts.

**Definition 7 (see [20).**

Let (*L*, ∨, ∧,^*c*^, 0,1) be a fuzzy lattice. If the dual mappings $\underline{apr}:L\to L$ and $\bar{apr}:L\to L$ satisfy the following conditions:

 (P1) $\underline{apr}(\alpha )={\left(\bar{apr}(\alpha^{c})\right)}^{c}\;\;(\forall \alpha \in L)$,
 (P2) $\underline{apr}(1)=1$,
 (P3) $\underline{apr}(\alpha \wedge \beta )=(\underline{apr}\alpha )\wedge (\underline{apr}\beta )\;\;(\forall \alpha ,\beta \in L)$,
 (P4) $\alpha \leq \bar{apr}(\alpha ),\forall \alpha \in L$,

then $\left(L,\vee ,\wedge {,}^{c},0,1,\underline{apr},\bar{apr}\right)$ is called a Pawlak algebra. $\bar{apr}$ (resp., $\underline{apr}$, pair ($\underline{apr},\bar{apr}$) ) is called upper approximation operator (resp., lower approximation operator, dual approximation operator) on *L*. If $\underline{apr}(\alpha )=\bar{apr}(\alpha )$, then *α* is called a definable element. If $\underline{apr}\alpha \neq \bar{apr}\alpha$, then *α* is called a rough element.

If (P3) is replaced by the following condition (P3)∗:

 (P3)∗$\alpha \leq \beta \Rightarrow \underline{apr(}\alpha )\leq \underline{apr}(\beta )$,

then $\bar{apr}$ (resp., $\underline{apr}$, pair ($\underline{apr},\bar{apr}$)) is called a weak upper approximation operator (resp., weak lower approximation operator, weak dual approximation operator) on *L*, and the system $\left(L,\vee ,\wedge {,}^{c},0,1,\underline{apr},\bar{apr}\right)$ is called a weak Pawlak algebra.

Let (*L*, ∨, ∧,^*c*^, 0,1) be a fuzzy lattice. Denote by *APR*(*L*) (resp., *APR*_*W*_(*L*)) the set of all dual approximation operators (resp., dual weak approximation operators) on *L*, respectively, and by *σ*_0_^*apr*^(*L*) the set of all definable elements in the Pawlak algebra $\left(L,\vee ,\wedge {,}^{c},0,1,\underline{apr},\bar{apr}\right)$.

**Definition 8 .**

Let (*L*, ∨, ∧,^*c*^, 0,1) be a fuzzy lattice and $\left({\underline{apr}}_{1},{\bar{apr}}_{1}\right)$, $\left({\underline{apr}}_{2},{\bar{apr}}_{2})\in APR_{w}(L\right)$. Then the dual weak approximation operator $\left({\underline{apr}}_{2},{\bar{apr}}_{2}\right)$ is called* rougher* than $\left({\underline{apr}}_{1},{\bar{apr}}_{1}\right)$, denoted by $\left({\underline{apr}}_{1},{\bar{apr}}_{1})≺({\underline{apr}}_{2},{\bar{apr}}_{2}\right)$, if the following inequalities hold:

(2) $$\begin{array}{c} \forall \alpha \in L,\;{\underline{apr}}_{2}\left(\alpha \right)\leq {\underline{apr}}_{1}\left(\alpha \right)\leq {\bar{apr}}_{1}\left(\alpha \right)\leq {\bar{apr}}_{2}\left(\alpha \right). \end{array}$$

**Proposition 9 .**

Let $\left(L,\vee ,\wedge {,}^{c},0,1,\underline{apr},\bar{apr}\right)$ be a Pawlak algebra. Then *APR*(*L*)⊆*APR*_*w*_(*L*).

**Proof**

Let $\left(\underline{apr},\bar{apr})\in APR(L\right)$. For any *α*, *β* ∈ *L* with *α* ≤ *β*, we have *α* = *α*∧*β*, and so

(3) $$\begin{array}{c} \underline{apr}\left(\alpha \right)=\underline{apr}\left(\alpha \wedge \beta \right)=\underline{apr}\left(\alpha \right)\wedge \underline{apr}\left(\beta \right). \end{array}$$

Therefore, $\underline{apr}(\alpha )\leq \underline{apr}(\beta )$, implying that condition (P3)∗ holds. Hence $\left(\underline{apr},\bar{apr})\in APR_{W}(L\right)$, as required.

Define two dual approximation operators $I∶=(\underline{I},\bar{I})$ and $O∶=(\underline{O},\bar{O})$ as follows:

(4) $$\begin{array}{c} \forall \alpha \in L,\;\underline{I}\left(\alpha \right)=\{\begin{array}{cc} 0 & \alpha \neq 1\\ 1 & \alpha =1 \end{array},\;\bar{I}\left(\alpha \right)=\{\begin{array}{cc} 0 & \alpha =0\\ 1 & \alpha \neq 0 \end{array}\\ \forall \alpha \in L,\;\underline{O}\left(\alpha \right)=\bar{O}\left(\alpha \right)=\alpha . \end{array}$$

Then it is easy to see that *I*, *O* ∈ *APR*(*L*) and $O≺(\underline{apr},\bar{apr})≺I$ for any $\left(\underline{apr},\bar{apr})\in APR_{w}(L\right)$.

It is possible to characterize *APR*_*W*_(*L*) according to the following result.

**Theorem 10 .**

Let (*L*, ∨, ∧,^*c*^, 0,1) be a fuzzy lattice. Then (*APR*_*W*_(*L*), ≺) is a complete distributive lattice with maximum element *I* and minimum element *O*.

**Proof**

Suppose that ${\left\{\left({\underline{apr}}_{t},{\bar{apr}}_{t}\right)\right\}}_{t\in T}\subseteq APR_{w}(L)$, where *T* is an index set. Define ${⋁}_{t\in T}{\underline{apr}}_{t}$ by

(5) $$\begin{array}{c} \left(\underset{t\in T}{⋁}{\underline{apr}}_{t}\right)\left(\alpha \right)=\underset{t\in T}{⋁}{\underline{apr}}_{t}\left(\alpha \right),\;\forall \alpha \in L. \end{array}$$

And ${⋀}_{t\in T}{\underline{apr}}_{t}$, ${⋁}_{t\in T}{\bar{apr}}_{t}$, and ${⋀}_{t\in T}{\bar{apr}}_{t}$ can be similarly defined. Then the following assertions hold.

 (1) Consider $\left({⋁}_{t\in T}{\underline{apr}}_{t},{⋀}_{t\in T}{\bar{apr}}_{t}\right)$, $\left({⋀}_{t\in T}{\underline{apr}}_{t},{⋁}_{t\in T}{\bar{apr}}_{t})\in APR_{w}(L\right)$. In fact, for any *α*, *β* ∈ *L*, by Definitions 6 and 7, we have
   (P1) $\left({⋁}_{t\in T}{\underline{apr}}_{t})(\alpha \right)$ = ${⋁}_{t\in T}{\underline{apr}}_{t}(\alpha )$ = ${⋁}_{t\in T}{\left({\bar{apr}}_{t}(\alpha^{c})\right)}^{c}$ = ${\left({⋀}_{t\in T}{\bar{apr}}_{t}(\alpha^{c})\right)}^{c}$ = ${\left(({⋀}_{t\in T}{\bar{apr}}_{t})(\alpha^{c})\right)}^{c}$,
   (P2) $({⋁}_{t\in T}{\underline{apr}}_{t})(1)={⋁}_{t\in T}{\underline{apr}}_{t}(1)=1$,
   (P3) if *α* ≤ *β*, then ${\underline{apr}}_{t}(\alpha )\leq {\underline{apr}}_{t}(\beta )$, ∀*t* ∈ *T*, implying that ${⋁}_{t\in T}{\underline{apr}}_{t}(\alpha )\leq {⋁}_{t\in T}{\bar{apr}}_{t}(\beta )$,
   (P4) $\alpha \leq {⋀}_{t\in T}{\bar{apr}}_{t}(\alpha )=({⋀}_{t\in T}{\bar{apr}}_{t})(\alpha )$ since $\alpha \leq {\bar{apr}}_{t}(\alpha )$ for all *t* ∈ *T*.
 (2) Consider $\left({⋀}_{t\in T}{\underline{apr}}_{t},{⋁}_{t\in T}{\bar{apr}}_{t})\in APR_{w}(L\right)$. The proof is analogous to that of (1).
 (3) Consider $\inf\left\{({\underline{apr}}_{t},{\bar{apr}}_{t})|t\in T\right\}=({⋁}_{t\in T}{\underline{apr}}_{t},{⋀}_{t\in T}{\bar{apr}}_{t})$ and $\sup\left\{({\underline{apr}}_{t},{\bar{apr}}_{t})|t\in T\right\}$ = $\left({⋀}_{t\in T}{\underline{apr}}_{t},{⋁}_{t\in T}{\bar{apr}}_{t}\right)$. Clearly, $\left({\underline{apr}}_{t},{\bar{apr}}_{t}\right)$ *≺* $\left({⋁}_{t\in T}{\underline{apr}}_{t},{⋀}_{t\in T}{\bar{apr}}_{t}\right)$ is true for all *t* ∈ *T*. In fact, let $\left(\underline{apr},\bar{apr})\in APR_{w}(L\right)$ be such that $\left(\underline{apr},\bar{apr})≺({\underline{apr}}_{t},{\bar{apr}}_{t}\right)$ for all *t* ∈ *T*. Then ${\underline{apr}}_{t}(\alpha )\leq \underline{apr}(\alpha )\leq \bar{apr}(\alpha )\leq {\bar{apr}}_{t}(\alpha )$ for all *t* ∈ *T* and *α* ∈ *L* by definition. It follows that
  (6) $$\begin{array}{c} \underset{t\in T}{⋁}{\underline{apr}}_{t}\left(\alpha \right)\leq \underline{apr}\left(\alpha \right)\leq \bar{apr}\alpha \leq \underset{t\in T}{⋀}{\bar{apr}}_{t}\left(\alpha \right),\;\forall \alpha \in L. \end{array}$$

On the other hand, it is obvious that $\left({\underline{apr}}_{t},{\bar{apr}}_{t})≺({⋁}_{t\in T}{\underline{apr}}_{t},{⋀}_{t\in T}{\bar{apr}}_{t}\right)$ for all *t* ∈ *T*. Hence, $\inf\left\{({\underline{apr}}_{t},{\bar{apr}}_{t})|t\in T\right\}=({⋁}_{t\in T}{\underline{apr}}_{t},{⋀}_{t\in T}{\bar{apr}}_{t})$. In a similar way by duality, we have $\sup\left\{({\underline{apr}}_{t},{\bar{apr}}_{t})|t\in T\right\}=({⋀}_{t\in T}{\underline{apr}}_{t},{⋁}_{t\in T}{\bar{apr}}_{t})$.

Summing up the above analysis, (*APR*_*W*_(*L*), ≺) is a complete lattice. It is obvious that *I* is the maximum element and *O* is the minimum element and that (APR_*W*_(*L*), ≺) is distributive. This completes the proof.

**Proposition 11 .**

Let (*L*, ∨, ∧,^*c*^, 0,1) be a fuzzy lattice and (*L*, *δ*) a lattice topology space. Define the operators ${\underline{apr}}_{\delta }:L\to L$ and ${\bar{apr}}_{\delta }:L\to L$ by

(7) $$\begin{array}{c} {\underline{apr}}_{\delta }\left(\alpha \right)=\vee \left\{\gamma \;|\;\gamma \leq \alpha ,\gamma \in \delta \right\},\\ {\bar{apr}}_{\delta }\left(\alpha \right)=\wedge \left\{\gamma^{c}\;|\;\alpha \leq \gamma^{c},\gamma \in \delta \right\}, \end{array}$$

respectively, for all *α* ∈ *L*. Then the system $\left(L,\vee ,\wedge {,}^{c},0,1,{\underline{apr}}_{\delta },{\bar{apr}}_{\delta }\right)$ is a Pawlak algebra.

**Proof**

It is straightforward and omitted.

**Proposition 12 .**

Let $\left(L,\vee ,\wedge {,}^{c},0,1,\underline{apr},\bar{apr}\right)$ be a Pawlak algebra. Then (*L*, *σ*_0_^*apr*^(*L*)) is a zero-dimensional lattice topology space.

**Proof**

It is evident that $\sigma_{0}^{apr}(L)=\left\{\alpha \in L|\alpha =\underline{apr}(\alpha )\right\}\cap \left\{\alpha \in L|\alpha =\bar{apr}(\alpha )\right\}$. And we conclude that the following assertions hold.

- $\left\{\alpha \in L|\alpha =\underline{apr}(\alpha )\right\}$ is union-closed. Suppose that $\left\{\alpha_{t}|t\in T\right\}\subseteq \left\{\alpha \in L|\alpha =\underline{apr}(\alpha )\right\}$, where *T* is an index set. Now, for any *s* ∈ *T*, we have *α*_*s*_ ≤ ⋁_*t*∈*T*_ *α*_*t*_, and so $\alpha_{s}=\underline{apr}(\alpha_{s})\leq \underline{apr}({⋁}_{t\in T}\alpha_{t})$, implying that ${⋁}_{t\in T}\alpha_{t}\leq \underline{apr}({⋁}_{t\in T}\alpha_{t})$. Since ⋁_*t*∈*T*_ *α*_*t*_ ≥ $\underline{apr}({⋁}_{t\in T}\alpha_{t})$, we have ${⋁}_{t\in T}\alpha_{t}=\underline{apr}({⋁}_{t\in T}\alpha_{t})$ by conditions (P1) and (P4), as required.
- $\left\{\alpha \in L|\alpha =\bar{apr}(\alpha )\right\}$ is intersection-closed. Suppose that $\left\{\alpha_{t}|t\in T\right\}\subseteq \left\{\alpha \in L|\alpha =\bar{apr}(\alpha )\right\}$, where *T* is an index set. For any *s* ∈ *T*, we have *α*_*s*_ ≥ ⋀_*t*∈*T*_ *α*_*t*_, and so $\alpha_{s}=\underline{apr}(\alpha_{s})\geq \bar{apr}({⋀}_{t\in T}\alpha_{t})$, implying that ${⋀}_{t\in T}\alpha_{t}\geq \bar{apr}({⋀}_{t\in T}\alpha_{t})$. By condition (P4), we have ${⋀}_{t\in T}\alpha_{t}=\bar{apr}({⋀}_{t\in T}\alpha_{t})$, as required.
- $\left\{\alpha \in L|\alpha =\underline{apr}(\alpha )\right\}\cap \left\{\alpha \in L|\alpha =\bar{apr}(\alpha )\right\}$ is complement-closed. For any $\alpha \in \left\{\alpha \in L|\alpha =\underline{apr}(\alpha )\right\}$ *∩* $\left\{\alpha \in L|\alpha =\bar{apr}(\alpha )\right\}$, by condition (P1), we have $\underline{apr}(\alpha^{c})$ = ${\left({\left(\underline{apr}(\alpha^{c})\right)}^{c}\right)}^{c}$ = ${\left(\bar{apr}(\alpha )\right)}^{c}=\alpha$, implying that *α* ^*c*^∈$\left\{\alpha |\alpha =\underline{apr}(\alpha )\right\}$ *∩* $\left\{\alpha |\alpha =\bar{apr}(\alpha )\right\}$. Hence $\left\{\alpha |\alpha =\underline{apr}(\alpha )\right\}\cap \left\{\alpha |\alpha =\bar{apr}(\alpha )\right\}$ is complement-closed.

Summing up the above analysis, (*L*, *σ*_0_^*apr*^(*L*)) is a zero-dimensional lattice topology space.

Now, let us turn our attention to the study of the transitive closure of approximation operators.

Let (*L*, ∨, ∧,^*c*^, 0,1) be a fuzzy lattice. Define the operator “∘” on *APR*(*L*) as follows:

(8) $$\begin{array}{c} \left({\underline{apr}}_{1},\bar{apr_{1}}\right)\circ \left({\underline{apr}}_{2},\bar{apr_{2}}\right)∶=\left({\underline{apr}}_{1}\circ {\underline{apr}}_{2},{\bar{apr}}_{1}\circ {\bar{apr}}_{2}\right). \end{array}$$

That is,

(9) ∀α∈L, (apr_1,apr1¯)∘(apr_2,apr2¯)(α) =(apr_1(apr_2(α)),apr¯1(apr¯2(α))),

where $\left({\underline{apr}}_{1},\bar{apr_{1}}),({\underline{apr}}_{2},\bar{apr_{2}})\in APR(L\right)$. For any positive integer *k*, define ${\left(\underline{apr},\bar{apr}\right)}^{\left(k+1\right)}∶={\left(\underline{apr},\bar{apr}\right)}^{\left(k\right)}\circ (\underline{apr},\bar{apr})$. Denote $\mathrm{cl}(\underline{apr},\bar{apr})∶={⋁}_{k=1}^{\infty }{\left(\underline{apr},\bar{apr}\right)}^{\left(k\right)}$, which is called the* transitive closure* of $\left(\underline{apr},\bar{apr}\right)$. Then, we obtain the following result.

**Lemma 13 .**

Let (*L*, ∨, ∧,^*c*^, 0,1) be a fuzzy lattice. Then

1. $\left({\underline{apr}}_{1},{\bar{apr}}_{1})≺({\underline{apr}}_{1},{\bar{apr}}_{1})\circ ({\underline{apr}}_{2},{\bar{apr}}_{2}\right)$ for all $\left({\underline{apr}}_{1},{\bar{apr}}_{1}),({\underline{apr}}_{2},{\bar{apr}}_{2})\in APR(L\right)$;
2. $\left({\underline{apr}}_{1},{\bar{apr}}_{1})\circ ({\underline{apr}}_{2},{\bar{apr}}_{2})\in APR(L\right)$;
3. $\mathrm{cl}(\underline{apr},\bar{apr})$ is a dual approximation operator.

**Proof**

(1) It is straightforward and omitted.

(2) It suffices to prove that $\left({\underline{apr}}_{1},{\bar{apr}}_{1})\circ ({\underline{apr}}_{2},{\bar{apr}}_{2}\right)$ satisfies conditions (P1)–(P4) in Definition 7. In fact, for any *α*, *β* ∈ *L*, we have

 (P1) ${\underline{apr}}_{1}({\underline{apr}}_{2}(\alpha ))={\bar{apr}}_{1}{\left({\left({\underline{apr}}_{2}(\alpha )\right)}^{c}\right)}^{c}=({\bar{apr}}_{1}{\left({\left({\bar{apr}}_{2}{\left(\alpha^{c})\right)}^{c}\right)}^{c}\right)}^{c}={\left({\bar{apr}}_{1}\left({\bar{apr}}_{2}\left(\alpha^{c}\right)\right)\right)}^{c}$,
 (P2) $({\underline{apr}}_{1}\circ {\underline{apr}}_{2})(1)={\underline{apr}}_{1}({\underline{apr}}_{2}(1))={\underline{apr}}_{1}(1)=1$,
 (P3) $\left({\underline{apr}}_{1}\circ {\underline{apr}}_{2})(\alpha \wedge \beta \right)$ = ${\underline{apr}}_{1}({\underline{apr}}_{2}(\alpha \wedge \beta ))$ = ${\underline{apr}}_{1}({\underline{apr}}_{2}(\alpha )\wedge {\underline{apr}}_{2}(\beta ))$ = $\left({\underline{apr}}_{1}({\underline{apr}}_{2}(\alpha ))\right)$ ∧ $\left({\underline{apr}}_{1}({\underline{apr}}_{2}(\beta ))\right)$ = $\left({\underline{apr}}_{1}\circ {\underline{apr}}_{2})(\alpha )\right)$ ∧ $\left({\underline{apr}}_{1}\circ {\underline{apr}}_{2})(\beta )\right)$,
(P4) ∀*α* ∈ *L*, $\alpha \leq {\bar{apr}}_{2}(\alpha )\Rightarrow \alpha \leq {\bar{apr}}_{1}(\alpha )\leq {\bar{apr}}_{1}({\bar{apr}}_{2}(\alpha ))=({\bar{apr}}_{1}\circ {\bar{apr}}_{2})(\alpha )$.

(3) From the proof of Theorem 10, we have

(10) $$\begin{array}{c} \mathrm{cl}\left(\underline{apr},\bar{apr}\right)=\left(\overset{\infty }{\underset{k=1}{⋀}}{\underline{apr}}^{\left(k\right)},\overset{\infty }{\underset{k=1}{⋁}}{\bar{apr}}^{\left(k\right)}\right). \end{array}$$

And it is easy to prove by mathematical induction that

(11) apr_(k)(α∧β) =apr_(k)(α)∧apr_(k)(β) for  all  positive  integer  k.

Therefore,

(12) (⋀k=1∞apr_(k))(α∧β)=⋀k=1∞apr_(k)(α∧β)=⋀k=1∞(apr_(k)(α)∧apr_(k)(β))=(⋀k=1∞apr_(k)(α))∧(⋀k=1∞apr_(k)(β)).

It follows that $\mathrm{cl}(\underline{apr},\bar{apr})$ satisfies condition (P3). Similarly, we can prove that $\mathrm{cl}(\underline{apr},\bar{apr})$ also satisfies conditions (P1), (P2), and (P4). Hence, $\mathrm{cl}(\underline{apr},\bar{apr})$ is a dual approximation operator.

**Theorem 14 (see [21]).**

If $\left(L,\vee ,\wedge {,}^{c},0,1,\underline{apr},\bar{apr}\right)$ is a Pawlak algebra, then the subset $T_{apr}=\left\{\gamma |\underline{apr}(\gamma )=\gamma \right\}$ is a lattice topology on *L*.

**Theorem 15 .**

Let (*L*, ∨, ∧,^*c*^, 0,1) be a fuzzy lattice and $\left(\underline{apr},\bar{apr})\in APR(L\right)$. Then for any *α* ∈ *L*, one has

(13) $$\begin{array}{c} \left(\overset{\infty }{\underset{k=1}{⋀}}{\underline{apr}}^{\left(k\right)}\right)\left(\alpha \right)=\vee \left\{\gamma \;|\;\gamma \leq \alpha ,\underline{apr}\left(\gamma \right)=\gamma \right\}, \end{array}$$

(14) $$\begin{array}{c} \left(\overset{\infty }{\underset{k=1}{⋁}}{\bar{apr}}^{\left(k\right)}\right)\left(\alpha \right)=\wedge \left\{\tau \;|\;\alpha \leq \tau ,\bar{apr}\left(\tau \right)=\tau \right\}. \end{array}$$

**Proof**

It suffices to prove (13). Equation (14) can be proved by the duality of approximation operators. By the infinite-union-closing in *T*_*apr*_, for any *α* ∈ *L*, we have

(15) apr_[∨{γ ∣ γ≤α,apr_(γ)=γ}] =∨{γ ∣ γ≤α,apr_(γ)=γ}≤α,apr_[∨{γ ∣ γ≤α,apr_(γ)=γ}] =∨{γ ∣ γ≤α,apr_(γ)=γ}≤apr_(α),apr_[∨{γ ∣ γ≤α,apr_(γ)=γ}] =∨{γ ∣ γ≤α,apr_(γ)=γ}≤apr_(2)(α)⋮apr_[∨{γ ∣ γ≤α,apr_(γ)=γ}] =∨{γ ∣ γ≤α,apr_(γ)=γ}≤apr_(k)(α),⋮

implying that $\vee \left\{\gamma |\gamma \leq \alpha ,\underline{apr}(\gamma )=\gamma \right\}\leq ({⋀}_{k=1}^{\infty }{\underline{apr}}^{\left(k\right)})(\alpha )$. Conversely, for any *α* ∈ *L*,

(16) $$\begin{array}{c} \underline{apr}\left(\left(\overset{\infty }{\underset{k=1}{⋀}}{\underline{apr}}^{\left(k\right)}\right)\left(\alpha \right)\right)=\left(\overset{\infty }{\underset{k=1}{⋀}}{\underline{apr}}^{\left(k\right)}\right)\left(\alpha \right)\leq \alpha . \end{array}$$

It follows that $({⋀}_{k=1}^{\infty }{\underline{apr}}^{\left(k\right)})(\alpha )\in T_{apr}$, and so $({⋀}_{k=1}^{\infty }{\underline{apr}}^{\left(k\right)})(\alpha )\leq \vee \left\{\gamma |\gamma \leq \alpha ,\underline{apr}(\gamma )=\gamma \right\}$. Hence, (13) holds.

As a consequence of Theorem 15, we obtain the following result.

**Corollary 16 .**

If $\left(L,\vee ,\wedge {,}^{c},0,1,\underline{apr},\bar{apr}\right)$ is a Pawlak algebra, then ${\bar{apr}}_{*}∶={⋁}_{k=1}^{\infty }{\bar{apr}}^{\left(k\right)}$ is a closure operator on *L*.

## 4. The Relationships between Pawlak Algebra and Auxiliary Ordering

Gierz [19] introduces the concept of auxiliary ordering for the study of continuous lattice. In fact, the auxiliary ordering is an order relation which is rougher than the initial ordering and can be regarded as the approximation of initial ordering. Inspired by this idea, we can use approximation operator to describe order approximately.

The following results present the relationships between Pawlak algebra and strong auxiliary ordering.

**Theorem 17 .**

Let (*L*, ∨, ∧,^*c*^, 0,1) be a fuzzy lattice and “≺” a strong auxiliary ordering on *L*. Define two operators ${\underline{apr}}_{≺}:L\to L$ and ${\bar{apr}}_{≺}$::*L* → *L* as follows:

(17) $$\begin{array}{c} \forall \alpha \in L,\;{\underline{apr}}_{≺}\left(\alpha \right)∶=\vee \left\{\gamma \;|\;\gamma ≺\alpha ,\gamma \in L\right\},\\ {\bar{apr}}_{≺}\left(\alpha \right)={\left({\underline{apr}}_{≺}(\alpha^{c})\right)}^{c}; \end{array}$$

then $\left(L,\vee ,\wedge {,}^{c},0,1,{\underline{apr}}_{≺},{\bar{apr}}_{≺}\right)$ is a Pawlak algebra.

**Proof**

It is obvious that condition (P1) holds. In what follows, we prove that conditions (P2)–(P4) are satisfied.

(1) The strong auxiliary ordering ≺ implies that 1≺1 is true, and so we have
  (18) $$\begin{array}{c} {\underline{apr}}_{≺}\left(1\right)=\vee \left\{\gamma \;|\;\gamma ≺1,\gamma \in L\right\}=1, \end{array}$$
  implying that condition (P2) holds.
(2) Let *α*, *β*, *γ* ∈ *L* be such that *γ*≺*α*∧*β*. Then *γ* ≤ *γ*≺*α*∧*β* ≤ *α* and *γ* ≤ *γ*≺*α*∧*β* ≤ *β*. By condition (ii) in Definition 1, we have *γ*≺*α*, *γ*≺*β*, and so
  (19) $$\begin{array}{c} \left\{\gamma \;|\;\gamma ≺\alpha \wedge \beta ,\gamma \in L\right\}\subseteq \left\{\gamma \;|\;\gamma ≺\alpha ,\gamma \in L\right\},\\ \left\{\gamma \;|\;\gamma ≺\alpha \wedge \beta ,\gamma \in L\right\}\subseteq \left\{\gamma \;|\;\gamma ≺\beta ,\gamma \in L\right\}. \end{array}$$

Therefore

(20) $$\begin{array}{c} \vee \left\{\gamma \;|\;\gamma ≺\alpha \wedge \beta ,\gamma \in L\right\}\leq \vee \left\{\gamma \;|\;\gamma ≺\alpha ,\gamma \in L\right\},\\ \vee \left\{\gamma \;|\;\gamma ≺\alpha \wedge \beta ,\gamma \in L\right\}\leq \vee \left\{\gamma \;|\;\gamma ≺\beta ,\gamma \in L\right\}. \end{array}$$

It follows that ${\underline{apr}}_{≺}(\alpha \wedge \beta )\leq {\underline{apr}}_{≺}(\alpha )$ and ${\underline{apr}}_{≺}(\alpha \wedge \beta )\leq {\underline{apr}}_{≺}(\beta )$, and hence

(21) $$\begin{array}{c} {\underline{apr}}_{≺}\left(\alpha \wedge \beta \right)\leq \left({\underline{apr}}_{≺}\left(\alpha \right)\right)\wedge \left({\underline{apr}}_{≺}\left(\beta \right)\right). \end{array}$$

On the other hand, by condition (iii) in Definition 3, we have

(22) $$\begin{array}{c} \vee \left\{\gamma \;|\;\gamma ≺\alpha ,\gamma \in L\right\}≺\alpha ,\\ \vee \left\{\gamma \;|\;\gamma ≺\beta ,\gamma \in L\right\}≺\beta . \end{array}$$

It follows from

(23) (∨{γ ∣ γ≺α,γ∈L}) ∧(∨{γ ∣ γ≺β,γ∈L})≤∨{γ ∣ γ≺α,γ∈L}≺α≤α,(∨{γ ∣ γ≺α,γ∈L}) ∧(∨{γ ∣ γ≺β,γ∈L})≤∨{γ ∣ γ≺β,γ∈L}≺β≤β

that

(24) $$\begin{array}{c} \left(\vee \left\{\gamma \;|\;\gamma ≺\alpha ,\gamma \in L\right\}\right)\wedge \left(\vee \left\{\gamma \;|\;\gamma ≺\beta ,\gamma \in L\right\}\right)≺\alpha ,\\ \left(\vee \left\{\gamma \;|\;\gamma ≺\alpha ,\gamma \in L\right\}\right)\wedge \left(\vee \left\{\gamma \;|\;\gamma ≺\beta ,\gamma \in L\right\}\right)≺\beta . \end{array}$$

Hence

(25) $$\begin{array}{c} \left(\vee \left\{\gamma \;|\;\gamma ≺\alpha ,\gamma \in L\right\}\right)\wedge \left(\vee \left\{\gamma \;|\;\gamma ≺\beta ,\gamma \in L\right\}\right)≺\alpha \wedge \beta , \end{array}$$

implying that

(26) (∨{γ ∣ γ≺α,γ∈L}) ∧(∨{γ ∣ γ≺β,γ∈L})∈{γ ∣ γ≺α∧β,γ∈L}.

Thus ${\underline{apr}}_{≺}(\alpha )\wedge {\underline{apr}}_{≺}(\beta )\leq {\underline{apr}}_{≺}(\alpha \wedge \beta )$, and so ${\underline{apr}}_{≺}(\alpha )\wedge {\underline{apr}}_{≺}(\beta )={\underline{apr}}_{≺}(\alpha \wedge \beta )$. Hence condition (P3) holds.

(3) By the duality of approximation operators, it suffices to prove that ${\underline{apr}}_{≺}(\alpha )\leq \alpha$. And this is clearly true by the definition of ${\underline{apr}}_{≺}(\alpha )$.

Summing up the above statements, $\left(L,\vee ,\wedge {,}^{c},0,1,{\underline{apr}}_{≺},{\bar{apr}}_{≺}\right)$ is a Pawlak algebra.

**Theorem 18 .**

Let $\left(L,\vee ,\wedge {,}^{c},0,1,\underline{apr},\bar{apr}\right)$ be a Pawlak algebra. Define a binary relation “≺” as follows:

(27) $$\begin{array}{c} \forall \alpha ,\beta \in L,\;\alpha ≺\beta ⟺\bar{apr}\left(\alpha \right)\leq \beta . \end{array}$$

Then “≺” is an auxiliary ordering on *L*.

**Proof**

(1) Let *α*, *β* ∈ *L*be such that *α*≺*β*. Then *α* ≤ *β* since $\alpha \leq \bar{apr}(\alpha )$ and $\bar{apr}(\alpha )\leq \beta$.

(2) Let *α*, *β*, *η*, *λ* ∈ *L* be such that *η* ≤ *α*≺*β* ≤ *λ*. Then $\bar{apr}(\eta )\leq \bar{apr}(\alpha )$ and $\bar{apr}(\alpha )\leq \beta \leq \lambda$. It follows that $\bar{apr}(\eta )\leq \lambda$; that is, *η*≺*λ*.

(3) Let *α*, *β*, *λ* ∈ *L* be such that *α*≺*γ* and *β*≺*γ*. Then $\bar{apr}(\alpha )\leq \gamma$ and $\bar{apr}(\beta )\leq \gamma$. Thus,

(28) $$\begin{array}{c} \bar{apr}\left(\alpha \vee \beta \right)=\bar{apr}\left(\alpha \right)\vee \bar{apr}\left(\beta \right)\leq \gamma , \end{array}$$

implying that *α*∨*β*≺*γ*.

(4) It follows from $0=\bar{apr}(0)\leq \alpha$ that 0≺*α* for all *α* ∈ *L*.

Summing up the above statements, “≺” is an auxiliary ordering on *L*.

## 5. Molecular Pawlak Algebra and Rough Topology

In this section, we focus on the approximate structure on fuzzy lattices. This structure can be regarded as abstract system of rough set.

**Definition 19 .**

Let $\left(L(\pi ),\vee ,\wedge {,}^{c},\underline{apr},\bar{apr}\right)$ be a molecular Pawlak algebra, (*D*, ≥) a directed set, and {*S*(*d*)}_*d*∈*D*_ a molecular net and *α* ∈ *π*. If there exists *d*_0_ ∈ *D* such that $\underline{apr}(\alpha )\leq S(d)\leq \bar{apr}(\alpha )$ for any *d* ≥ *d*_0_, then *α* is called a* rough limit* of *S*, denoted by $S\overset{apr}{\to }\alpha$. The set of all rough limits of *S* is denoted by lim⁡*S*.

In the sequel, we provide two examples of molecular Pawlak algebra in topology space.

**Example 20 .**

Let (*U*, *ρ*) be a metric space, *L* = Φ(*U*), and *π* = {{*x*}∣*x* ∈ *U*}, where Φ(*U*) denotes the set of all subsets of *U*. Define

(29) $$\begin{array}{c} \forall A\in L,\;\bar{apr}A∶=\underset{x\in A}{⋃}\bar{B}\left(x,1\right),\;\underline{apr}A∶={\left(\bar{apr}A^{c}\right)}^{c}, \end{array}$$

where $\bar{B}(x,1)$ is the closed ball. Then $\left(\Phi \left(U\right)\left(\pi \right),\cup ,\cap {,}^{c},\underline{apr},\bar{apr}\right)$ is a molecular Pawlak algebra. Let *x*_0_ be the *apr*-limit of the molecular sequence {*x*_*n*_}_*n*∈*N*_ (i.e., $x_{n}\overset{apr}{\to }x_{0}$), which means that there exists *n*_0_ ∈ *N* such that *ρ*(*x*_*n*_, *x*_0_) ≤ 1 for *n* ≥ *n*_*o*_. Obviously, $x_{n}\to x_{0}\;\;\text{implies}\;\;x_{n}\overset{apr}{\to }x_{0}\;\;(n\to \infty )$.

**Example 21 .**

Let *U* be a nonempty set, *R* an equivalent relation on *U*, *L* = *F*(*U*) fuzzy power set on *U*, *π* = {{*x*_*λ*_}∣*x* ∈ *U*, *λ* ∈ (0,1]}, and *U*/*R* = {*X*_*i*_}_*i*∈*I*_. For any *A* ∈ *F*(*U*), define two fuzzy sets *F* ^*A*^ and *F*_*A*_ on *U*/*R* as

(30) $$\begin{array}{c} \forall i\in I,\;F^{A}\left(X_{i}\right)=\sup\left\{A\left(x\right)\;|\;x\in X_{i}\right\},\\ F_{A}\left(X_{i}\right)=\inf\left\{A\left(x\right)\;|\;x\in X_{i}\right\}. \end{array}$$

Then we have, for all *A*, *B* ∈ *F*(*U*),

1. *F* ^*A*∪*B*^ = *F* ^*A*^ ∪ *F* ^*B*^,
2. *F*_*A*∩*B*_ = *F*_*A*_∩*F*_*B*_,
3. *F* ^*A*∩*B*^⊆*F* ^*A*^∩*F* ^*B*^,
4. *F*_*A*∪*B*_⊇*F*_*A*_ ∪ *F*_*B*_,
5. *F*_*A*_⊆*F* ^*A*^.

Moreover, for any *A* ∈ *F*(*U*), define $\bar{apr}A$ and $\underline{apr}A$ as

(31) $$\begin{array}{c} \left(\bar{apr}A\right)\left(x\right)=\{\begin{array}{cc} F^{A}\left(X_{i}\right) & x\in X_{i}\\ 0 & x\notin X_{i}, \end{array} \end{array}$$

(32) $$\begin{array}{c} \left(\underline{apr}A\right)\left(x\right)=\{\begin{array}{cc} F_{A}\left(X_{i}\right) & x\in X_{i}\\ 0 & x\notin X_{i}, \end{array} \end{array}$$

for any *x* ∈ *U*. Then $\left(F\left(U\right)\left(\pi \right),\cup ,\cap {,}^{c},\underline{apr},\bar{apr}\right)$ is a molecular Pawlak algebra.

In the system $\left(F\left(U\right)\left(\pi \right),\cup ,\cap {,}^{c},\underline{apr},\bar{apr}\right)$, the rough neighborhood $\bar{apr}(x_{\lambda })$ of molecule *x*_*λ*_ is represented as

(33) $$\begin{array}{c} \bar{apr}\left(x_{\lambda }\right)\left(y\right)=\{\begin{array}{cc} \lambda & R\left(x,y\right)=1\\ 0 & R\left(x,y\right)=0, \end{array} \end{array}$$

$S\overset{apr}{\to }x_{\lambda }$ if and only if there exists *d*_0_ ∈ *D* such that *xRy* ^*d*^ and *λ*_*d*_ ≤ *λ* for *d* ≥ *d*_0_, where *S*(*d*) = *y*_*λ*_*d*__^*d*^ and *X*_*i*_ is poly-point set.

If *U*/*R* = {{*x*}∣*x* ∈ *U*}, namely, *R*(*x*, *y*) = 1 if and only if *x* = *y*, then we have

(34) $$\begin{array}{c} \bar{apr}\left(x_{\lambda }\right)=\underline{apr}\left(x_{\lambda }\right)=x_{\lambda } \end{array}$$

for fuzzy point *x*_*λ*_ and it is know that $S\overset{apr}{\to }x_{\lambda }$ if and only if there exists *d*_0_ ∈ *D* such that *S*(*d*) = *x*_*λ*_ for *d* ≥ *d*_0_.

**Definition 22 .**

A molecular net is called *R*-*fuzzy-rough-convergent* if and only if it is *apr*-convergent in the molecular Pawlak algebra $\left(F\left(U\right)\left(\pi \right),\cup ,\cap {,}^{c},\underline{apr},\bar{apr}\right)$ defined by formulas (31) and (32).

The following is now straightforward.

**Proposition 23 .**

The *R*-fuzzy-rough-convergent classes satisfy the Moore-Smith conditions can induce a topology, called *R*-rough fuzzy topology, which is a nullity topology with square members.

**Theorem 24 .**

Let (*L*(*π*), ∨, ∧,^*c*^, 0,1) be a molecular lattice. Then any mapping *h* : *π* → *L* satisfying *α* ≤ *h*(*α*) can at least induce a molecular Pawlak algebra.

**Proof**

Set $\bar{h}(0)=0$, and define

(35) $$\begin{array}{c} \bar{h}\left(\gamma \right)∶=\underset{\alpha \leq \gamma ,\alpha \in \pi }{⋁}h\left(\alpha \right),\;\;\underline{h}\left(\gamma \right)={\left(\bar{h}\left(\gamma^{c}\right)\right)}^{c}. \end{array}$$

Then it is evident that $\left(L(\pi ),\vee ,\wedge {,}^{c},\underline{h},\bar{h}\right)$ is a molecular Pawlak algebra.

## 6. The Application of Approximation Operators to Rough Sets

Yao and Lin [16] introduce the concept of rough sets with general binary relation. They defined *R*-neighborhood of *x* from a universe *U* by the binary relation *R* on *U*,

(36) $$\begin{array}{c} r\left(x\right)∶=\left\{y\;|\;\left(x,y\right)\in R\right\}, \end{array}$$

and defined general dual approximation operators ${\underline{apr}}_{R}$ and ${\bar{apr}}_{R}$ as, for all *X*⊆*U*,

(37) $$\begin{array}{c} {\underline{apr}}_{R}\left(X\right)∶=\left\{x\;|\;r\left(x\right)\subseteq X\right\},\\ {\bar{apr}}_{R}\left(X\right)∶=\left\{x\;|\;r\left(x\right)\cap X\neq ⌀\right\}. \end{array}$$

In this section, we further investigate the properties of approximation operators induced by a binary relation.

The following results recall some basic properties of approximation operators induced by a binary relation.

**Proposition 25 (see [16]).**

Let *R*⊆*U* × *U* be a similar relation on *U*. Then the following assertions hold: for all *x*, *y* ∈ *U* and *X*, *Y*⊆*U*,

(R1) ${\underline{apr}}_{R}(X)={\left[{\bar{apr}}_{R}(X^{c})\right]}^{c}$, ${\bar{apr}}_{R}(X)={\left[{\underline{apr}}_{R}(X^{c})\right]}^{c}$;
(R2) ${\underline{apr}}_{R}(⌀)=\bar{apr_{R}}(⌀)=⌀$, ${\underline{apr}}_{R}(U)={\bar{apr}}_{R}(U)=U$;
(R3) ${\underline{apr}}_{R}(X\cap Y)={\underline{apr}}_{R}(X)\cap {\underline{apr}}_{R}(Y)$, ${\bar{apr}}_{R}(X\cap Y)\subseteq {\bar{apr}}_{R}(X)\cap {\bar{apr}}_{R}(Y)$;
(R4) ${\underline{apr}}_{R}(X\cup Y)⊇{\underline{apr}}_{R}(X)\cup {\underline{apr}}_{R}(Y)$, ${\bar{apr}}_{R}(X\cup Y)={\bar{apr}}_{R}(X)\cup {\bar{apr}}_{R}(Y)$;
(R5) ${\underline{apr}}_{R}(X)\subseteq X\subseteq {\bar{apr}}_{R}(X)$;
(R6) $x\in {\bar{apr}}_{R}\{y\}\Leftrightarrow y\in {\bar{apr}}_{R}\{x\}$.

Proposition 25 indicates that the system $\left(\Phi (U),\cup ,\cap {,}^{c},⌀,U,{\underline{apr}}_{R},{\bar{apr}}_{R}\right)$ is a Pawlak algebra, where Φ(*U*) denotes the set of all subsets of *U*.

**Proposition 26 (see [22]).**

Let *R*⊆*U* × *U* be a binary relation on *U*. Then

1. *R* is reflexive if and only if ${\underline{apr}}_{R}(X)\subseteq X\subseteq {\bar{apr}}_{R}(X)\;\;(\forall X\subseteq U)$;
2. *R* is symmetric if and only if $x\in {\bar{apr}}_{R}\{y\}$ is equivalent to $y\in {\bar{apr}}_{R}\{x\}$;
3. the reflexive relation *R* is transitive if and only if  ${\bar{apr}}_{R}$ is a closure operator.

**Proposition 27 .**

Let $\left(\underline{p},\bar{p})\in APR(\Phi (U)\right)$, where $\bar{p}$ is a closure operator. Then

(38) $$\begin{array}{c} x\in \bar{p}\left\{y\right\}\;iff\;\;\bar{p}\left\{x\right\}\subseteq \bar{p}\left\{y\right\}. \end{array}$$

**Proof**

It is straightforward and omitted.

**Theorem 28 .**

Let ($\Phi \left(U\right),\cup ,\cap {,}^{c},⌀,U,\underline{p},\bar{p})$ be a Pawlak algebra, where $\bar{p}$ is a closure operator that satisfies (P6) for all $x,y\in U,x\in \bar{p}\{y\}\Leftrightarrow y\in \bar{p}\{x\}$. Then we have the following:

(1) the binary relation *R*_*p*_ defined as
  (39) $$\begin{array}{c} R_{p}∶=\left\{\left(x,y\right)\;|\;x\in \bar{p}\left\{y\right\}\right\} \end{array}$$
  is an equivalent relation on *U*;
(2) $\left({\underline{apr}}_{R_{p}},{\bar{apr}}_{R_{p}})≺(\underline{p},\bar{p}\right)$. Moreover, if *U* is a finite universe or the cover ${\left\{\bar{p}\{x\}\right\}}_{x\in U}$ of *U* is finite, then $\left({\underline{apr}}_{R_{p}},{\bar{apr}}_{R_{p}})=(\underline{p},\bar{p}\right)$.

**Proof**

(1) It follows from Proposition 29 and condition (P6) that

(40) $$\begin{array}{c} R_{p}=\left\{\left(x,y\right)\;|\;\bar{p}\left\{x\right\}=\bar{p}\left\{y\right\}\right\}. \end{array}$$

Then it is easy to see that *R*_*p*_ is an equivalent relation, where ${\left[x\right]}_{R}=\bar{p}\{x\}$ for all *x* ∈ *U*.

(2) For any *X*⊆*U*, we have

(41) apr¯Rp(X)={x ∣ [x]Rp∩X≠⌀}={x ∣ {y ∣ x∈p¯{y}}∩X≠⌀}={x ∣ ∃y∈X  such  that  x∈p¯{y}}⊆p¯(X).

On the other hand, ${\bar{apr}}_{R_{p}}(X^{c})\subseteq \bar{p}(X^{c})$ implies that ${\left(\bar{p}(X^{c})\right)}^{c}\subseteq {\left({\bar{apr}}_{R_{p}}(X^{c})\right)}^{c}$; hence $\underline{p}(X)\subseteq {\underline{apr}}_{R_{p}}(X)$. Thus, we have $\left({\underline{apr}}_{R_{p}},{\bar{apr}}_{R_{p}})≺(\underline{p},\bar{p}\right)$.

Suppose that universe *U* is finite and let *X* = {*y*_1_, *y*_2_,…, *y*_*m*_}⊆*U*. Then we have

(42) p¯(X)=⋃yk∈Xp¯{yk}={x ∣ ∃y∈X  such  that  x∈p¯{y}}={x ∣ ∃y∈X  such  that  y∈[x]Rp}={x ∣ [x]Rp∩X≠⌀}=apr¯Rp(X).

Analogous to the above proof, we have $\underline{p}(X)={\underline{apr}}_{R_{p}}(X)$. It follows that $\left({\underline{apr}}_{R_{p}},{\bar{apr}}_{R_{p}})=(\underline{p},\bar{p}\right)$.

Now suppose that the cover ${\left\{\bar{p}\{x\}\right\}}_{x\in U}$ of  *U* is finite; that is, there exist *x*_*k*_ ∈ *U*, *k* = 1,2,…, *m* such that ${\left\{\bar{p}\{x_{k}\}\right\}}_{k\in \{1,2,\ldots ,m\}}$ is a cover of *U*. Analogous to the above proof, to prove that $\left({\underline{apr}}_{R_{p}},{\bar{apr}}_{R_{p}})=(\underline{p},\bar{p}\right)$, it suffices to prove that, for all *X*⊆*U*, $\bar{p}(X)\subseteq {\bar{apr}}_{R}(X)$. In fact, it follows from ${\left\{\bar{p}\{x_{k}\}\right\}}_{k\in \{1,2,\ldots ,m\}}$ being a cover of *U* that $X\subseteq U\subseteq {⋃}_{k=1}^{m}\bar{p}\{x_{k}\}$. Hence

(43) p¯(X)⊆p¯(⋃k=1mp¯{xk})=⋃k=1mp¯{xk}={x ∣ there  exists  xk∈X  such  that  x∈p¯{xk}}={x ∣ there  exists  xk∈X  such  that  p¯{x}=p¯{xk}}={x ∣ there  exists  xk∈X  such  that  xk∈[x]Rp}={x ∣ [x]Rp∩X≠⌀}=apr¯Rp(X),

as required.

In the sequel, denote by *R* the set of all similar relations on *U*. Then it is evident that *R* is infinite-intersection-closed. And the following result holds.

**Proposition 29 .**

Let *R* be the set of all similar relations on *U*. Then we have

1. [*x*]_⋂_*t*∈*T*_*R*_*t*__ = ⋂_*t*∈*T*_[*x*]_*R*_*t*__ for {*R*_*t*_}_*t*∈*T*_⊆*R*, where *T* is an index set;
2. ∀*R*_1_, *R*_2_ ∈ *R*, $R_{1}\subseteq R_{2}\Leftrightarrow ({\underline{apr}}_{R_{1}},{\bar{apr}}_{R_{1}})≺({\underline{apr}}_{R_{2}},{\bar{apr}}_{R_{2}})$.

**Proof**

(1) Consider [*x*]_⋂_*t*∈*T*_*R*_*t*__ = {*y*∣(*x*, *y*) ∈ ⋂_*t*∈*T*_ *R*_*t*_} = {*y*∣∀ *t* ∈ *T*, (*x*, *y*) ∈ *R*_*t*_} = ⋂_*t*∈*T*_{*y*∣(*x*, *y*) ∈ *R*_*t*_} = ⋂_*t*∈*T*_[*x*]_*R*_*t*__.

(2) Let *R*_1_, *R*_2_ ∈ *R* be such that *R*_1_⊆*R*_2_. Then for any *x* ∈ *U*, we have [*x*]_*R*_1__⊆[*x*]_*R*_2__. Now, let *X*⊆*U*. If $x\in {\underline{apr}}_{R_{2}}(X)$, then [*x*]_*R*_2__⊆*X*, implying that [*x*]_*R*_1__ = [*x*]_*R*_1_∩*R*_2__ = [*x*]_*R*_1__∩[*x*]_*R*_2__⊆[*x*]_*R*_2__⊆*X*; that is, $x\in {\underline{apr}}_{R_{1}}(X)$. Therefore, ${\underline{apr}}_{R_{2}}(X)\subseteq {\underline{apr}}_{R_{1}}(X)$ and ${\bar{apr}}_{R_{1}}(X)\subseteq {\bar{apr}}_{R_{2}}(X)$ by the duality. It follows that $\left({\underline{apr}}_{R_{1}},{\bar{apr}}_{R_{1}})≺({\underline{apr}}_{R_{2}},{\bar{apr}}_{R_{2}}\right)$.

Conversely, suppose that $\left({\underline{apr}}_{R_{1}},{\bar{apr}}_{R_{1}})≺({\underline{apr}}_{R_{2}},{\bar{apr}}_{R_{2}}\right)$ and (*x*, *y*) ∈ *R*_1_. It follows that $x\in {\bar{apr}}_{R_{1}}\{y\}$. Since ${\bar{apr}}_{R_{1}}(X)\subseteq {\bar{apr}}_{R_{2}}(X)$ for any *X*⊆*U* by the assumption, we know that ${\bar{apr}}_{R_{1}}\{y\}\subseteq {\bar{apr}}_{R_{2}}\{y\}$, and hence $x\in {\bar{apr}}_{R_{2}}\{y\}$, implying that (*x*, *y*) ∈ *R*_2_. Therefore, *R*_1_⊆*R*_2_.

**Theorem 30 .**

Let *U* be a finite set and *R* a similar relation on *U*. Then there exists an equivalent relation $\bar{R}$ such that $\mathrm{cl}({\underline{apr}}_{R},{\bar{apr}}_{R})=({\underline{apr}}_{\bar{R}},{\bar{apr}}_{\bar{R}})$ and that $\bar{R}$ is the transitive closure of *R*.

**Proof**

It follows from the proof of Theorem 10 that $\mathrm{cl}({\underline{apr}}_{R},{\bar{apr}}_{R})=({⋀}_{k=1}^{\infty }{{\underline{apr}}_{R}}^{\left(k\right)},{⋁}_{k=1}^{\infty }{{\bar{apr}}_{R}}^{\left(k\right)})$ and that ${⋁}_{k=1}^{\infty }{{\bar{apr}}_{R}}^{\left(k\right)}$ is a closure operator satisfying (R6). Now, we define a binary relation $\bar{R}$ on *U* as follows:

(44) $$\begin{array}{c} \bar{R}∶=\left\{\left(x,y\right)\;|\;x\in \left(\overset{\infty }{\underset{k=1}{⋃}}{{\bar{apr}}_{R}}^{\left(k\right)}\right)\left\{y\right\}\right\}. \end{array}$$

Then, it is evident that $\bar{R}$ is a similar relation on *U* and $\mathrm{cl}({\underline{apr}}_{R},{\bar{apr}}_{R})=({\underline{apr}}_{\bar{R}},{\bar{apr}}_{\bar{R}})$ by Theorem 10. In the sequel, we prove that $\bar{R}$ is the transitive closure of *R*, which also implies that $\bar{R}$ is an equivalent relation on *U*.

Suppose that R* is an equivalent relation such that *R*⊆R*. By Proposition 29, we have $\left({\underline{apr}}_{R},{\bar{apr}}_{R})≺({\underline{apr}}_{R^{*}},{\bar{apr}}_{R^{*}}\right)$ and ${\bar{apr}}_{R^{*}}$ is a closure operator, and hence $\left({\underline{apr}}_{\bar{R}},{\bar{apr}}_{\bar{R}})≺({\underline{apr}}_{R^{*}},{\bar{apr}}_{R^{*}}\right)$. Thus, by Proposition 29, $\bar{R}\subseteq R^{*}$.

## 7. Conclusions

In this paper, we have investigated the general approximation structure, weak approximation operators, and Pawlak algebra in the framework of fuzzy lattice, lattice topology, and auxiliary ordering. The relationships between the Pawlak approximation structures and these mathematic structures are established, and some related properties are presented. These works would provide a new direction for the study of rough set theory and information systems. As for future research, it will be interesting to continue the study of molecular Pawlak algebra and general partial approximation spaces.
